# What you see is what you (don't) get: a comment on Funke's (2014) opinion paper

**DOI:** 10.3389/fpsyg.2014.01120

**Published:** 2014-10-16

**Authors:** Samuel Greiff, Romain Martin

**Affiliations:** Education, Culture, Cognition and Society Unit, Institute of Cognitive Science and Assessment, University of LuxembourgLuxembourg, Luxembourg

**Keywords:** complex problem solving, problem solving, causal cognition, measurement, microworlds, minimal complex systems

In an opinion paper published in this journal, Funke (2014) argues that two different types of assessment instruments for complex problem solving (CPS), computer-simulated microworlds (CSMs), and minimal complex systems (MCSs), might require different types of causal cognition. CPS denotes the ability to successfully deal with new, intransparent, and dynamically changing problem situations (Funke, 2001) and is considered one of the most important skills of the 21^st^ century. Given the recent attention CPS has received from both academic and educational stances, for instance, through the Programme for International Student Assessment, which tested CPS in 15-year-old students across more than 40 countries (OECD, 2014), the topic addressed by Funke (2014) is both timely and of high relevance.

In this commentary, we will elaborate on the difference between specific CPS assessment instruments used in a variety of research fields (e.g., experimental psychology, educational assessment) and CPS as the underlying attribute, and we will offer a view that diverges from Funke's (2014). We will express our hope that different CPS assessment traditions that are reflected in CSMs and MCSs will converge toward a generalizable understanding of CPS as an unobserved latent attribute (i.e., a psychological concept) that is of relevance to researchers from a number of fields including experimental psychology and individual differences research. Before alluding specifically to Funke's opinion paper, we would like to specify our terminology. Funke uses the terms CPS and MCS for the two types of assessment. However, CPS denotes a psychological attribute and not a specific set of assessment instruments. Thus, we will use the established terms *computer-simulated microworlds (CSMs)* and *minimal complex systems (MCSs)* for the assessment instruments, and we will reserve the term *complex problem solving (CPS)* for the latent attribute that both types of assessments claim to tap into.

Funke (2014) argues that the usefulness of CSMs and MCSs for measuring CPS depends on the field of study. CSMs, with their realistic and highly complex setup, their many interrelated variables, and their knowledge-rich semantic embedding, are best applied in experimental settings, whereas MCSs, with an analytical approach that is geared toward reducing complexity and a high level of standardization, are best suited for (educational) assessment purposes. This position is reflected in the predominant use of CSMs in experimental and cognitive research (e.g., Dörner, 1980) and of MCSs in (educational) assessment (e.g., Wüstenberg et al., 2012). On the basis of task analyses, Funke claims that the type of causal cognition, the heuristics, and the strategies required in CSMs and MCSs differ substantially and, thus, do not allow for direct comparisons between the two.

For any study, it is of crucial importance that the employed measures—be they CSMs or MCSs—tap into the construct they claim to capture even though the measures may differ with regard to their difficulty or their surface features. Admittedly, CSMs and MCSs look quite different at first sight. CSMs are knowledge-rich and simulate complex real-world scenarios such as business companies (e.g., the Tailorshop) or entire cities (e.g., Lohhausen), whereas MCSs are knowledge-lean and less complex. But can we really conclude, on the basis of purely conceptual task analyses, that they tap into different types of causal cognition? And if so, what is it that they tap into? 15 years ago, Süß (1999) had already provided important empirical guidance on this question with regard to the construct validity of CSMs. He showed that the performances in three different CSMs were moderately correlated—which was to be expected given that they supposedly all measured CPS. More importantly, when controlling for fluid intelligence and specific prior knowledge, the correlation dropped to non-significance. In the terms of contemporary theories on the human intellect (McGrew, 2009), the shared variance of three CSMs originated from fluid intelligence and (specific) prior knowledge. That is, the type of causal cognition required to successfully master CSMs is empirically identical to the causal cognition required for standard tests of fluid intelligence. Provocatively, one could state that the Tailorshop, probably the best-known microworld included in Süß's study, requires nothing but fluid intelligence and inductive reasoning as well as specialized knowledge about how companies work. Following this, the strong impact of context in CSMs and the substantial advantages for participants who possess this specific knowledge are not only assets of CSMs that make them more realistic but also disadvantages that distort the measurement of the underlying attribute and the cognitive processes associated with it. In addition, the high complexity of CSMs (sometimes up to 1,000 variables) does not allow for a complete causal analysis—an asset according to Funke. However, this may also lead to unsystematic variance created by the fact that subjects have to deal with an environment that is basically unpredictable for them. That is, CSMs may produce unsystematic variance because they are just *too* complex and leave participants with no choice but to either guess or apply some general reasoning skills that are also found in classical intelligence and reasoning tests. And neither guessing nor abstract reasoning are distinctive features that are found in contemporary definitions of CPS as unique characteristics. Thus, despite their high face validity, empirical evidence suggests that CSMs may fall short of tapping into the type of causal cognition unique to CPS or causal cognition at all—a point not mentioned in Funke's (2014) paper.

Indeed, reports such as the one by Süß (1999) have led to a notable decline in the number of studies on CPS using CSMs in the late 1990s and the early 2000s because they questioned the empirical usefulness of CSMs and the existence of CPS as a latent attribute. It was argued that CSMs were unable to provide any evidence suggesting that they tapped into other than already well-known and established attributes such as reasoning and prior knowledge. At the same time, efforts were undertaken to solve the aforementioned issues by introducing more formalized CPS assessments that focused on the core features of CPS such as dynamics, complexity, and intransparency and that tried to minimize the impact of unsystematic and construct-unrelated variance (e.g., Funke, 2001; Kröner et al., 2005). One of these efforts cumulated in the development of MCSs (e.g., Greiff et al., 2013).

Obviously, avoiding any unnecessary ballast in favor of a focus on the core attribute of CPS comes at a cost: severe reductions in face validity. MCSs lack the appealing and attractive real-world resemblance of CSMs, which constitute a much more prosaic assessment environment. At the same time, we should ask whether the complex contextual embedding of CSMs has ever been shown to allow valid conclusions about participants' abilities to act efficiently in the real-world context simulated by CSMs. In this regard too, results are rather mixed because experience and expertise with the real-word context do not necessarily warrant better decisions in CSMs (but see also Putz-Osterloh and Lemme, 1987; Chapman et al., 2006; Elliott et al., 2007). To this end, we do agree with Funke (2014) that MCSs lack the appeal of the complex real-world problem situations that are found in CSMs, but we respectfully disagree with his notion that MCSs capture a type of causal cognition that does not require the use of heuristics (Greiff et al., 2013) or of sophisticated strategies (Neubert et al., in press).

Interestingly, the use of strategies and heuristics relates directly to the question of cultural differences in CPS. In one of the few studies addressing this topic, Strohschneider and Güss (1999) report that different cultural backgrounds have an impact on the type of strategy and heuristics used in CSMs. It is important to understand whether such cultural differences originate from different prior knowledge (e.g., the kind of business knowledge needed in the Tailorshop might vary a great deal across cultures) or whether they constitute genuine differences in the underlying cognitive processes of CPS (e.g., it might be that culturally different conventions impact the way problems are approached). To this end, data on the processes taking place in CPS derived from computer-generated log files provide a useful tool for further penetrating the actual behavioral correlates in both CSMs and MCSs. For instance, Wittmann and Hattrup (2004) used log file analyses to show that boys tend to outperform girls in the Tailorshop because of a lower level of risk aversiveness reflected through more and stronger interventions in the system, which led, in turn, to better problem solutions.

An abundance of research questions await answers on how cultural background might influence the cognitive processes that occur when people tackle complex problems. But here again, we should ask whether these scientific challenges can be better addressed with CSMs, which are related to a real-world embedding almost necessarily bound to the cultural context of the real-world environment that is simulated or with the more context-free and perhaps more culture-free instruments provided by MCSs. In building up further knowledge on CPS, we will need a clear distinction between face validity on the one hand and the underlying CPS attribute and its defining characteristics on the other. Valid CPS assessments that serve the purpose of researchers from different fields, whether in the form of CSMs or MCSs, should be developed along the lines suggested by Borsboom et al. (2004) who state that “a test is valid for measuring an attribute if and only if (a) the attribute exists and (b) variations in the attribute causally produce variations in the outcomes of the measurement procedure” (p. 1061). For CPS assessments, both questions remain unanswered for the time being. Empirical rigor and scrutiny are needed more than anything else so that the construct validity of CPS measurements can be guaranteed, implying a process that cannot be driven mainly by the desire for face validity, however appealing this facet might be. Herein, we do not see a dissociation between experimental- and assessment-oriented studies but a potential synthesis that jointly works toward an understanding of CPS as a latent attribute beyond its specific assessment instruments.

## Author note

This research was funded by a grant from the Fonds National de la Recherche Luxembourg (ATTRACT “ASKI21”).

### Conflict of interest statement

The authors declare that the research was conducted in the absence of any commercial or financial relationships that could be construed as a potential conflict of interest.
